# A comparative study between intravenous and oral alendronate administration for the treatment of osteoporosis

**DOI:** 10.1186/s40064-015-1474-9

**Published:** 2015-11-05

**Authors:** Akira Horikawa, Naohisa Miyakoshi, Yoichi Shimada, Yusuke Sugimura, Hiroyuki Kodama

**Affiliations:** South Akita Orthopedic Clinic, Seiwakai, 96-2 Kaidousita, Syowa-Ookubo, Katagami, 018-1401 Japan; Department of Orthopedic Surgery, Akita University Graduate School of Medicine, 1-1-1 Hondo, Akita, 010-8543 Japan

## Abstract

It has recently been reported that bisphosphonates are the most common treatment for osteoporotic patients. However, they are many problems, including poor bioavailability and adherence, as well as adverse drug reactions. Therefore, intravenous administration of bisphosphonates has been developed to resolve these problems. In Japan today, alendronate and ibandronate have been approved for intravenous administration, and they have advantages, such as good adherence and better gastrointestinal tolerability, compared to oral administration. We attempted to confirm the effects of administration of intravenous alendronate, which is not inferior to oral administration, for osteoporotic patients in earlier research. 200 consecutive Japanese over 70 years-old postmenopausal women who visited the first author’s orthopedic clinic and had femoral neck or lumbar spine bone mineral density (BMD) values more than 2.5 SD lower than the reference values were randomly enrolled in this study. 100 subjects were recruited for administration of intravenous alendronates because of their poor adherence, no respond of treatment status, and gastrointestinal adverse effects. Furthermore, 10 of these subjects were excluded due to discontinuation, and a total of 90 subjects were eligible for the intravenous group. The remaining 50 patients received oral alendronate. The present study also showed no significant difference between intravenous and oral administration with respect to BMD, biochemical bone turnover markers, and the incidence of fractures. These results show that intravenous administration of alendronate is not inferior to oral alendronate for the treatment of osteoporosis. Therefore, intravenous administration of alendronate can be recommended if patients do not tolerate or adhere to oral bisphosphonates.

## Background

Although it has been widely recognized that alendronate (ALN) is the drug of first choice for the treatment of osteoporosis, its adverse effects with oral intake are an issue. To address these problems, intravenous bisphosphonate regimens have been developed. In Japan today, alendronate and ibandronate have been approved for intravenous administration for the treatment of osteoporosis. A randomized, double-masked, comparative study was initiated to examine the safety of intravenous administration of alendronate in a clinical trial in Japanese patients with osteoporosis (Shiraki et al. 2012). However, few comparative studies to confirm the effectiveness of intravenous administration of alendronate have been conducted in patients with osteoporosis. Therefore, the primary objective of the present study was to confirm that intravenous administration of alendronate is not inferior to oral administration of alendronate for Japanese patients with osteoporosis.

## Methods

### Study design

This study was designed as a 52-week, prospective, non-randomized study involving parallel-group comparison between intravenous ALN 900 µg (alendronate sodium hydrate, Teijin Pharmaceutical Company, Tokyo, Japan) once monthly and oral ALN 35 mg (alendronate sodium hydrate, Teijin Pharmaceutical Company) once weekly. Both of these are almost same dosage (Huruhata et al. 2013).

Patients with poor adherence to oral administration and/or gastrointestinal problems were assigned to receive intravenous drip infusion of ALN (intravenous drip group), while the others were assigned to oral ALN administration (oral group). All patients gave their informed consent prior to the study. This study was performed in accordance with the ethical standards laid down in the 1964 Declaration of Helsinki and its amendments.

### Participants

**Fig. 1:**
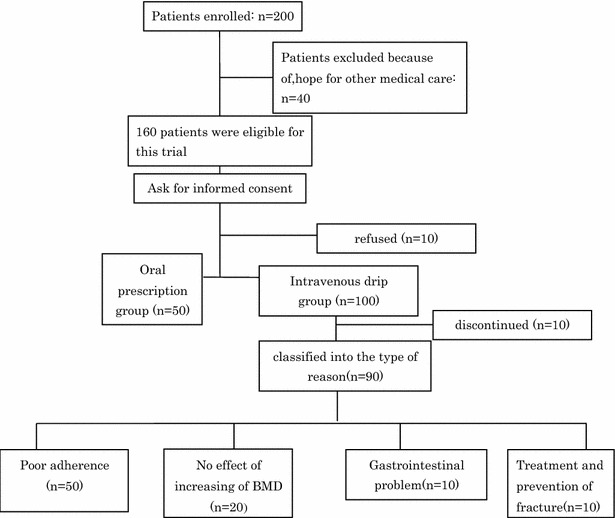
The participant selection process. Data on age, height, weight, BMI, and incidences of fracture and adverse drug reactions were collected from all participants

A total of 200 consecutive Japanese over 70 years-old postmenopausal women who visited the first author’s orthopedic clinic between March 2013 and April 2014 and had femoral neck or lumbar spine bone mineral density (BMD) values more than 2.5 SD lower than the reference values were randomly enrolled in this study. Of these, those who preferred other medical care and refused to provide their informed consent were excluded from this trial. The remaining 150 eligible patients were recruited. Of these, 100 subjects were recruited for administration of intravenous alendronates because of their poor adherence, no respond of treatment status, and gastrointestinal adverse effects. Those who complained of gastrointestinal (GI) problems want to try to other administration therapy which was similar alendronate. Furthermore, 10 of these subjects were excluded due to discontinuation, and a total of 90 subjects were eligible for the intravenous group. The remaining 50 patients received oral alendronate (Fig. 1).

### BMD measurements

Areal BMD measurements were performed at the proximal femur and lumbar spine using dual energy X-ray absorptiometry (DXA, Hologic QDR Discovery W type; Toyo Medic., Tokyo, Japan), and some of the patients who could not visit our clinic were examined by forearm BMD (DTX200; Datex DSM, Courtaboeuf Cedex, France, Japan) at a related facility.

### Fracture incidence rates and adverse events

Incidence rates of fractures, including vertebral and non-vertebral fractures, and adverse drug reactions (bone and muscle pain, pyrexia, myalgia, fatigue, lymphopenia, etc.) were calculated.

### Body mass index

Body mass index (BMI) was measured in all subjects who participated in this study.

### Bone turnover markers

Serum bone specific alkaline phosphatase (BAP) and serum collagen type 1 cross-linked N telopeptide (NTX) were measured at baseline, 6 months, and 12 months.

### Statistical analysis

Statistical analysis was performed using Microsoft Office Excel and the Statcel 3 program (OMS, Inc., Hyogo, Japan). Both BMD and bone turnover markers for each subject were analyzed by Student’s t test to compare differences between the two groups. For the incidences of fractures and adverse drug reactions, the Chi square test was used to evaluate the significance of differences. All results of statistical tests were regarded as significant with p < 0.05.

## Results

**Fig. 2:**
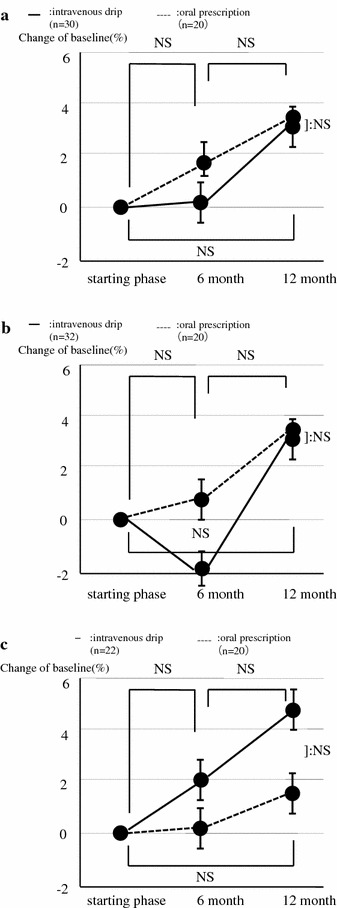
Statistical analysis of changes in bone mineral density between the oral group and the intravenous drip group (Student’s t test). These patients had no compliance about all kinds of BMD which were lumbar spine, femoral neck and forearm. Of these BMD, they choose only one examination area due to time and cost. Moreover, they had a few chance to measure their BMD due to lack of visit. **a** Lumbar spine, **b** femur, **c** forearm

**Fig. 3:**
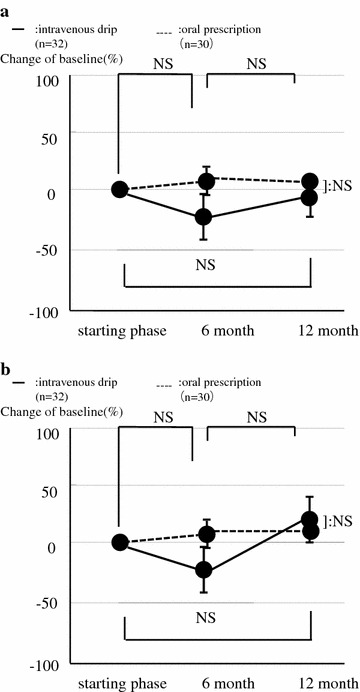
Comparison of changes in biochemical bone turnover markers between the oral group and the intravenous group (Student’s t test). I suppose bone turnover suppression were relatively low because of effect of other drug combination such as eldecalcitol or past oral alendronate prescription. **a** Bone alkaline phosphatase (BAP), **b** collagen type 1 cross-linked N telopeptide (NTX)

Data from the intravenous drip group and the oral prescription group are shown in Table 1. There were no significant differences in age, BMI, and the incidences of fractures and adverse drug reactions between these groups. Both of two new fractures were all vertebral fracture in intravenous drip group, of those which one was L1 and other was Th8. All of these patients visited twice a month after injury through 6 months. Decompression and deformity have not progressed during this period. Two adverse drug reactions were found in oral prescription group, all of those were pyrexia and they recovered soon. According to the DXA analysis, there was no difference in the change of BMD between these groups in the lumbar spine (oral group: 0.585, 0.595, 0.605, intravenous group: 0.552, 0.553, 0.568, sequentially) femur (oral group: 0.464, 0.467, 0.478, intravenous group: 0.491, 0.482, 0.506, sequentially) and forearm (oral group: 0.231, 0.232, 0.234, intravenous group: 0.256, 0.261, 0.269, sequentially), (Fig. 2a–c, g/cm^2^). There was also no significant difference in bone turnover markers between the intravenous group (BAP: 23.2, 16.3, 23.0, NTX: 20.7, 14.9, 21.3, sequentially) and the oral group (BAP: 21.5, 21.8, 21.6, NTx: 19.8, 20.1, 20.0, sequentially), (Fig. 3a, b, BAP: U/l, NTX: nmol BCE/l).

**Table 1:** Comparisons of baseline variables in intravenous drip and oral prescription group

	Oral prescription group (n = 50)	Intravenous drip group (n = 90)	p value
Age (years)	76 ± 7.9	80 ± 6.4	0.200^a^
Height (cm)	153 ± 4.2	150 ± 5.3	0.015^a^
Weight (kg)	54.8 ± 8.9	50.2 ± 7.6	0.023^a^
BMI (kg/m^2^)	25 ± 4.0	24.1 ± 3.6	0.201^a^
BMD: femur (g/cm^2^)	0.464 ± 0.035	0.491 ± 0.052	0.538^a^
BMD: lumbar spine (g/cm^2^)	0.585 ± 0.042	0.552 ± 0.028	0.052^a^
Previous fracture (n)	5	10	0.158^b^
Incidence ratio of fracture (n)	0	2	0.205^b^
Adverse drug reactions (n)	2	0	0.204^b^

^a^Student’s t test

^b^χ^2^ test

## Discussion

There have been only a few reports that compared the efficacy and safety of alendronate given intravenously once a month and orally once a week in Japanese patients with osteoporosis (Shiraki et al. 2012; Miyakoshi 2014). Therefore, the present study was performed to attempt to confirm the efficacy and safety of intravenous alendronate, which previous research has suggested is non-inferior to oral alendronate (Shiraki et al. 2012). Although this study was a limited developmental study with a short-duration (1 year) and a relatively small number of patients, we did not find any significant differences between these groups with regard to age, BMI, incidence ratio of previous fractures. So we confirm that there are no dysbalance about them. This study results also showed no inferiority of intravenous administration compared to oral administration in the changes in percentages of BMD from the baseline in the lumbar spine, femur, and forearm. Furthermore, the bone turnover markers (BAP and NTX) were changed to a similar level by the two treatments, and with respect to the safety of alendronate, no clinically significant differences in the incidences of fractures and adverse drug reactions were seen.

In addition, intravenous group seems to be low healthy because of GI problem, we could not find any other health problems without it. To take this condition into consideration, we regarded this group as having the same healthy condition.

Treatment of osteoporosis by intravenous infusion of bisphosphonates results in better bioavailability than by oral bisphosphonates, which was estimated at about 0.7 % (Gertz et al. 1995). The present data confirmed that the change in BMD was not significantly different between the two. These data may suggest that bioavailability is not important whether the patients are carefully instructed to follow the dosing guidance for oral medication or given intravenous administration.

On the other hand, intravenous infusion of bisphosphonates is known to induce a transient acute phase reaction with bone and muscle pain, pyrexia, myalgia, fatigue, lymphopenia, etc., and in some patients one might hesitate to prescribe oral alendronate because of issues with gastrointestinal tolerability, such as when there is kyphosis of the lumbar spine, which may be associated with gastroesophageal reflux disease (Miyakoshi et al. 2009). However, the above-mentioned adverse drug reactions did not occur in the present study.

Considering these facts and the lack of a significant difference in the incidence of fractures, the present study confirmed the efficacy and safety of intravenous alendronate 900 μg once a month, which were similar to those of weekly oral alendronate in Japanese patients with osteoporosis. Intravenous alendronate is considered to be useful instead of oral alendronate to achieve the expected effect of prevention of fractures if the patients have poor adherence for oral prescriptions (Brookhart et al. 2007; Siris et al. 2006).

## Conclusion

As previously reported, intravenous administration of alendronate is not inferior to oral alendronate for the treatment of osteoporosis. No significant differences were seen between the intravenous and oral alendronate groups with respect to changes in BMD, bone turnover markers, prevention of vertebral fractures, and adverse events. We need further investigation in long-term period and more number of patients in this study.
